# Untargeted plasma metabolomics based on liquid chromatography-mass spectrometry for the analysis of *Dendrobium officinale* on canine immunity and antioxidant status

**DOI:** 10.3389/fvets.2025.1642292

**Published:** 2025-09-29

**Authors:** Jie Yang, Xiaobing Yang, Baoguo Liu, Baiguan Shi, Lihong An, Dongtao Zhang, Qiaoxian Gao, Wenzhi Yang, Guosheng Xin

**Affiliations:** ^1^School of Life Sciences, NingXia University, Yinchuan, China; ^2^Key Lab of Ministry of Education for Protection and Utilization of Special Biological Resources in Western China, NingXia University, Yinchuan, China; ^3^School of Agriculture, Ningxia University, Yinchuan, China; ^4^Star Pet Kingdom (Beijing) Technology Co., Ltd., Beijing, China; ^5^Ningxia Haobiao Analysis and Test Institute, Yinchuan, China; ^6^College of Animal Science and Technology, Ningxia University, Yinchuan, China

**Keywords:** *Dendrobium officinale*, poodles, nutrient digestibility, blood biochemistry, plasma metabolomics

## Abstract

*Dendrobium officinale* Kimura et Migo (*D. officinale*) is a valuable traditional Chinese herb, rich in bioactive compounds like polysaccharides and flavonoids. It is recognized for its immunomodulatory and antioxidant properties. However, its impact on canine metabolic health remains unexplored. This study investigated the effects of *Dendrobium officinale* supplementation on nutrient digestibility, blood biochemical parameters, antioxidant activity, and serum metabolomics in domestic poodles. Thirty-two ((5.20 ± 0.26 kg)) healthy female poodles were randomly divided into four groups and fed diets containing 0% (control), 0.5%, 1%, or 2% *D. officinale* for 45 days. The results showed that *D. officinale* supplementation did not affect the apparent digestibility of dry matter (DM), crude protein (CP), crude fat (EE), crude ash (Ash), calcium (Ca) and phosphorus (P) in poodles (*p* > 0.05). The peak IgM concentration (1.04 g/L) was observed at a 1% supplementation level, while optimal TP and GLB levels (69.72 g/L and 34.67 g/L, respectively) were noted at 0.5% supplementation. The highest CAT activity (1.95 U/mL) and T-AOC (7.12 U/mL) were observed at a 1% level, while the highest GSH-Px activity (1630.56 U/mL) was recorded at 2% supplementation level. Metabolomic analysis identified ten significantly altered serum metabolites involved in oxidative stress, inflammatory mediation, and energy metabolism. Key metabolic pathways influenced included arachidonic acid, caffeine, pyruvate, and amino acid metabolism.These findings suggest that *D. officinale* enhances immune and antioxidant function in poodles without affecting nutrient digestion, likely through modulation of critical metabolic pathways. A supplementation level of 0.5% to 1% is recommended to achieve these beneficial effects.

## 1 Introduction

The significance of pet dogs as companion animals in human society is on the rise (1), prompting increased scrutiny of their well-being by researchers and the general populace (2). Enhancing pet health is now a primary objective in the advancement of functional additives for pet food (3). Chinese herbal medicine possesses the characteristics of naturalness and non-resistance, has demonstrated positive effects on gastrointestinal function, immune system, and disease prevention. This has led to a growing interest in Chinese herbal medicine as functional additives in pet food research (4). *D. officinale* is a traditional and valuable Chinese medicinal herb, contains various bioactive components such as phenols, polysaccharides, alkaloids, flavonoids, and other compounds (5). These components have been associated with diverse benefits including improved gastrointestinal function, anti-aging properties, and reduction of oxygen-free radicals (6). Research indicates that *Dendrobium* not only promotes digestion and absorption (7, 8), but also plays a crucial role in promoting health by boosting antioxidant capacity and supporting immune system function (9–12). *Dendrobium officinale* and its active components (e.g., dendrobium polysaccharides) have been shown to upregulate the expression of the antioxidant gene Nrf2, thereby promoting the expression of its downstream target genes. These include glutathione synthase catalytic subunit (GCLC) and Glutamate-cysteine ligase modifier subunit (GCLM), which are responsible for glutathione synthesis, ultimately elevating levels of multiple antioxidant enzymes in the bloodstream (10, 12). This molecular mechanism has been validated across various animal models: in mice, *Dendrobium* extract and polysaccharides enhance serum glutathione peroxidase (GSH-Px) activity and total antioxidant capacity (T-AOC) (13). In zebrafish models, it has been demonstrated to enhance superoxide dismutase (SOD) activity while effectively reducing malondialdehyde (MDA) and reactive oxygen species (ROS) levels, thereby improving oxidative stress status and exerting anti-aging effects (14). Furthermore, the active compounds present in Dendrobium regulate immune function through multiple pathways and targets, including the promotion of the proliferation and differentiation of immune cells, the activation of the complement system, and the modulation of cytokine release (15). Shan (16) and Huang et al. (17) demonstrated that *Dendrobium* polysaccharides stimulate the expression of immune factors and immunoglobulins (IgG and IgM), thereby fortifying the immune system in murine models. Wang (18) showed that *Dendrobium* polysaccharides modulate serum metabolites, including amino acids, glycerophospholipids, sphingolipids, and prostaglandins, to ameliorate liver injury through pathways involving glucose metabolism, amino acid metabolism, and lipid metabolism. These studies underscore the potential of *Dendrobium* as a functional additive. However, research on its utility as a supplement in pet food is scarce, and its effects on immune and antioxidant functions in canines, along with the underlying mechanisms, remain to be elucidated. Addressing this research gap, this study integrated *D. officinale* into the diet of poodles to assess its effects on nutrient digestibility, immunity, and antioxidant capacity across different supplementation levels. Furthermore, employing metabolomics techniques, the study compared alterations in small molecule in the dogs bloodstream before and after *D. officinale* supplementation. This methodology facilitated the detection of metabolite variances and the analysis of impacted pathways, offering a scientific appraisal of *D. officinale*’s influence on nutritional metabolism, physiological well-being, and other aspects of dog health. The outcomes seek to establish a theoretical foundation for utilizing *D. officinale* as a high-quality and dependable functional food additive in animal nutrition.

Plasma metabolomics facilitates the acquisition of dynamic and comprehensive insights into the body’s overall physiological state and pathological changes at specific time points through systematic analysis of numerous small-molecule metabolites in plasma. Liquid chromatography-mass spectrometry (LC–MS) is a highly sensitive and precise analytical technique that enables the systematic detection and quantification of low-abundance metabolites in plasma samples. This approach identifies metabolites with significant biological relevance and statistically significant differences, thereby elucidating metabolic processes and underlying mechanisms of change in biological systems (19).

## 2 Materials and methods

### 2.1 Animal ethics

This study was conducted at the experimental base of the Ningxia Feed Engineering Technology Research Center and was approved by the Animal Ethics Committee of Ningxia University (approval No. NXU-H-2023-093).

### 2.2 Materials

The *D. officinale* powder used in this research was sourced from the dried stems of Huoshan *D. officinale,* cultivated in a traditional Chinese medicine base located in Taishang Village, Qingshui Town, Mentougou District, Beijing. The preparation process involved initially crushing the dried stem segments with a crusher, followed by sieving through a 40-mesh standard sieve to obtain finely powdered material for experimentation. This powder has been verified to conform to the quality standards outlined in the Pharmacopeia of the People’s Republic of China (2022 edition) for *D. officinale.*

### 2.3 Experimental animals and experimental design

Thirty-two healthy adult female poodles, aged 1–2 years with similar body weights (5.20 ± 0.26 kg), were randomly divided into four groups, each consisting of eight poodles. The groups were designated as follows: Group A (control group, 0% *D. officinale*), Group B (0.5% *D. officinale*), Group C (1% *D. officinale*), and Group D (2% *D. officinale*). The study was conducted over a 45-day period, comprising a 15-day pre-test phase and a 30-day main test phase.

### 2.4 Experimental diet composition and nutritional level

The diet was formulated based on the Nutrition Requirements for Dogs and Cats (20) to fulfill the daily metabolic energy requirements of adult pet dogs, and the ingredients presented in Table 1.

**Table 1:** Composition and nutrient level of experimental diets (Dry matter basis).

Items	A	B	C	D
Chicken bone meal	5.00	5.03	5.06	5.16
Chicken powder	18.00	17.98	17.94	17.81
Chicken liver powder	5.00	4.99	4.96	4.96
Corn flour	42.00	41.40	40.74	39.57
Wheat flour	10.00	10.00	10.00	10.00
Oatmeal	6.00	6.10	6.30	6.50
Yeast extract	3.00	3.00	3.00	3.00
Chicken oil	4.00	4.00	4.00	4.00
Soybean oil	3.00	3.00	3.00	3.00
*D. officinale*	0.00	0.50	1.00	2.00
Premix	4.00	4.00	4.00	4.00
Total	100.0	100	100	100
Nutrient levels				
CP	25.98	25.98	25.97	25.97
EE	11.90	11.90	11.90	11.89
CF	1.82	1.90	1.97	2.13
Ash	3.89	3.91	3.92	3.96
Ca	0.75	0.75	0.75	0.75
P	0.94	0.94	0.94	0.94
ME, MJ/kg	18.30	18.28	18.25	18.20

Premix provided the following per kilogram of the diet: Fe 1,500 mg, Cu 300 mg, Zn 3,000 mg, Mn 240 mg, I 44 mg, Se 17.5 mg, Vitamin E 1500 IU, Vitamin A 227250 IU, Vitamin D3 27,600 IU, Vitamin B1 112.5 mg, Vitamin B3 850 mg, Vitamin B2 262.5 mg, Vitamin B6 75 mg, Vitamin B4 85 mg, Vitamin B12 1.75 mg, Ca 19,000 mg, Na 40,000 mg, Cl 60,000 mg, Mg 30,000 mg. A, dietary supplements 0% *D. officinale* in poodles (as control), B, dietary supplements 0.5% *D. officinale* in poodles, C, dietary supplements 1% *D. officinale* in poodles, D, dietary supplements 2% *D. officinale* in poodles; CP, crude protein; EE, ether extract; CF, crude fiber; Ash, crude ash; Ca, calcium; P, phosphorus; ME, metabolizable energy. The nutrient levels were calculated values.

### 2.5 Feeding management

The experimental poodles were individually housed in cages (1.25 m × 1.85 m). Prior to the trial, the kennels and cages underwent thorough cleaning and disinfection following standard protocols. The poodles were vaccinated and dewormed according to established procedures. The amount of food provided was calculated based on the daily maintenance metabolizable energy requirement (ME (kcal/d) = 140 × BW^0.75^) for young adult test dogs, which ME and BW, respectively, represent metabolizable energy and body weight. The poodles were fed twice daily at 08:00 and 17:00, with free access to water. A comprehensive observational approach was employed to monitor the diet intake, water consumption, incidence of diarrhea, mortality and excretion throughout the poodles feeding trial.

## 3 Sample collection and analytical determination

### 3.1 Fecal sample collection and processing

Fecal samples were collected using the total feces collection method in the last 7 days of the experiment. Fresh fecal sample were cleaned of hair and debris, weighed and placed in sample bags. Subsequently, the samples were mixed with 10% sulfuric acid and stored at −20 °C. Upon completion of fecal sample collection, the 7 days samples were combined and homogenized, then desiccated to a consistent weight at 105 °C. The desiccated samples were pulverized through a 40-mesh sieve and stored in sample bags for nutrient analysis.

### 3.2 Blood sample collection and processing

On the last day of the experiment, a blood sample was obtained from the anterior arm vein, Approximately 8 mL of blood into a vacuum blood collection tube without anticoagulants. The tube was then left at room temperature for about 3.5 h, before being centrifuged at 3,000 rpm for 10 min at 4 °C to isolate the serum. The resulting supernatant was then transferred to 1.5 mL centrifuge tubes and stored at −80 °C for subsequent analysis of serum biochemical and antioxidant parameters. Furthermore, blood samples were collected from four randomly selected poodles in each group, using blood collection tubes containing ethylene diamine tetraacetic acid (EDTA) as an anticoagulant. These samples were allowed to rest for 30 min at room temperature before undergoing the same processing procedure as the serum sample for blood metabolomics analysis.

### 3.3 Measurement indicators and methods

#### 3.3.1 Determination of apparent nutrient digestibility

Dietary and fecal routine nutrient composition was determined by the following procedures: the dry matter (DM) content was determined with reference to GB/T 6435-2014, the crude protein (CP) content was determined with reference to GB/T 6432-2018, the ether extract (EE) content was determined with reference to GB/T 6433-2006, the crude Ash (Ash) content was determined with reference to GB/T 6438-2007, the calcium (Ca) content was determined with reference to GB/T 6436-2018, and the phosphorus (P) content was determined with reference to GB/T 6437-2018. Apparent digestibility of nutrients was calculated by the following formula:

Apparent digestibility of nutrients (%) = [(nutrient intake–nutrient excretion) / nutrient intake] × 100.

#### 3.3.2 Determination of serum biochemical indices

The serum biochemical indices were quantified using the Snibe MAGLUMIX6 automatic chemiluminescence immunoassay system along with the corresponding reagents. The primary indicators measured included total cholesterol (TC), interferon-*γ* (γ-IFN), fasting glucose (GLU), total protein (TP), albumin (ALB), globulin (GLB), albumin/globulin ratio (A/G), total bilirubin (TBil), alanine aminotransferase (ALT), aspartate aminotransferase (AST), glutamyl transferase (GGT), direct bilirubin (DBIL), indirect bilirubin (IBIL), alkaline phosphatase (ALP), and total bile acids (TBA).

The serum immunoglobulin M (IgM) concentration was measured using an enzyme-linked immunosorbent assay (ELISA). The test kit was obtained from the Nanjing Jiancheng Bioengineering Research Institute (IgM; #H109-1-1, CV is 2.3%, Sensitivity is 0.5 g/L, detection range is 0.1–2.3 g/L), and the procedure was conducted in strict accordance with the manufacturer’s instructions.

#### 3.3.3 Determination of serum antioxidant indexes

Serum antioxidant markers: catalase (CAT; #A007-1-1, CV is 1.9%, sensitivity is 0.2 U/mL, detection range is 0.2–24.8 U/mL), malondialdehyde (MDA, #A003-1-2, CV is 2.3%, sensitivity is 0.5 nmoL/mL, detection range is 0.5–113.0 nmoL/mL), glutathione peroxidase (GSH-PX, #A005-1-2, CV is 3.56%, sensitivity is 20 U, detection range is 20-330 U) and total antioxidant capacity (T-AOC, #A015-1-2, CV is 3.60% sensitivity is 0.2 U/mL, detection range is 0.2–55.2 U/mL) were determined with the commercial test kit procured from Nanjing Jiancheng Biotechnology Research Institute (Nanjing, China), and the samples were processed and analyzed in accordance with the instructions provided with the kits.

#### 3.3.4 Determination of plasma metabolomics

The prepared plasma samples were sent to Wuhan Mavis Biotechnology Co. for plasma metabolomics analysis. The samples were thawed on ice and vortexed for 10 s to ensure thorough mixing. Subsequently, 50 μL of each sample was transferred to the corresponding Eppendorf tube, followed by the addition of 300 μL of a 20% acetonitrile-methanol internal standard extraction solution. The mixture was vortexed for 3 min. After centrifugation at 12,000 rpm for 10 min at 4 °C, 200 μL of the supernatant was transferred to a new, correspondingly numbered centrifuge tube and stored at −20 °C for 30 min. The samples were then centrifuged again at 12,000 rpm for 3 min at 4 °C, and 180 μL of the supernatant was transferred to a sample bottle for liquid chromatography–tandem mass spectrometry (LC–MS/MS) analysis. For metabolomic data analysis, Orthogonal Partial Least Squares Discriminant Analysis (OPLS-DA) was employed to investigate differences among groups. Differential metabolites were identified based on the variable importance in projection (VIP) score with a threshold of VIP score greater than 1, and fold change (FC) greater than 2.0 or less than 0.5, which were considered differentially expressed. The identified metabolites were annotated using Kyoto Encyclopedia of Genes and Genomes (KEGG) Compound database (http://www.kegg.jp/kegg/compound/), Subsequently, the annotated metabolites were mapped to the KEGG Pathway database (http://www.kegg.jp/kegg/pathway.html). Pathways with significantly regulated metabolites were then subjected to Metabolite Sets Enrichment Analysis (MSEA), their significance was determined by the *p*-values from the hypergeometric test.

### 3.4 Statistical analysis

Nutrient digestibility data, blood biochemical indices, and antioxidant parameters were initially organized using Excel 2019, followed by analysis through one-way analysis of variance (ANOVA) in SPSS version 24.0. Duncan’s multiple range test was employed for post-hoc comparisons, with differences deemed statistically significant at *p* < 0.05. The data are presented as mean ± standard deviation.

The correlation analysis between plasma metabolites and blood biochemical indices as well as antioxidant indices was conducted using Spearman correlation analysis.

## 4 Results

### 4.1 Nutrient metabolism

As shown in Table 2, the inclusion of *D. officinale* in the diet did not lead to significant changes in the digestibility of dry matter, crude protein, and ether extract. However, it had a considerable impact on the digestibility of ash (from 64.90 to 71.39%), Ca (from 40.40 to 51.03%), and P (from 45.97 to 56.99%), although these differences were not significant (*p* > 0.05), it indicated that *D. officinale* did not interfere with the absorption of dietary nutrients in poodles.

**Table 2:** Effects of *D. officinale* on nutrient apparent digestibility of poodles (*N* = 8). %.

Items	Groups				*p* value
	A	B	C	D	
DM	94.82 ± 0.33	94.66 ± 0.67	94.18 ± 0.37	95.17 ± 0.63	0.609
CP	93.52 ± 0.45	93.81 ± 0.74	93.15 ± 0.44	94.33 ± 0.77	0.592
EE	98.85 ± 0.08	98.78 ± 0.13	98.97 ± 0.07	99.11 ± 0.11	0.134
Ash	69.04 ± 2.10	65.58 ± 4.75	64.90 ± 2.01	71.39 ± 3.83	0.499
Ca	49.74 ± 4.50	46.13 ± 8.04	40.40 ± 3.36	51.03 ± 6.50	0.586
P	54.88 ± 3.37	48.58 ± 7.17	45.97 ± 3.04	56.99 ± 5.66	0.393

A, dietary supplements 0% *D. officinale* in poodles (as control); B, dietary supplements 0.5% *D. officinale* in poodles; C, dietary supplements 1% *D. officinale* in poodles; D, dietary supplements 2% *D. officinale* in poodles; DM, dry matter; CP, crude protein; EE, ether extract; Ash, crude ash; Ca, calcium; P, phosphorus.

Different letters (a, b, c) indicate statistical significance. Shoulder labels in peer data that did not contain the same letter indicated significant differences between these data *p* < 0.05, there was no significant difference between the same letters or no letters *p* > 0.05.

### 4.2 Serum biochemical indices

As shown in Table 3, incorporation of *D. officinale* into the diet significantly increased the levels of TP and GLB in the blood (*p* < 0.05). The trends TP and GLB are consistent, following the addition of *D. officinale,* the levels of both components are significantly elevated compared to the control group (*p* < 0.05). The peak TP and GLB levels were noted at the 0.5% supplementation level, reaching 69.72 g/L and 34.67 g/L, respectively. No significant effects were observed for the other indicators (*p* > 0.05).

**Table 3:** Effects *D. officinale* on serum biochemical indices of poodles (*N* = 8).

Items	Groups				*p* value
	A	B	C	D	
TC, mmol/L	4.87 ± 0.32	5.53 ± 0.60	5.29 ± 0.63	5.68 ± 0.65	0.808
GLU, mml/L	4.88 ± 0.13	4.20 ± 0.74	4.22 ± 0.51	4.41 ± 0.64	0.157
TP, g/L	63.77 ± 2.11^b^	69.72 ± 4.52^a^	69.56 ± 2.26^a^	68.65 ± 4.44^a^	0.026
ALB, g/L	34.25 ± 1.63	35.05 ± 1.01	36.01 ± 1.80	34.45 ± 1.81	0.251
GLB, g/L	29.52 ± 2.73^b^	34.67 ± 3.95^a^	33.55 ± 2.49^a^	34.20 ± 3.61^a^	0.048
A/G	1.17 ± 0.16	1.02 ± 0.11	1.08 ± 0.12	1.02 ± 0.11	0.154
TBil, umol/L	2.88 ± 0.38	3.25 ± 0.29	3.10 ± 0.37	3.31 ± 0.35	0.837
ALT, U/L	35.17 ± 2.50	43.75 ± 3.24	32.88 ± 4.30	30.63 ± 3.43	0.062
AST, U/L	34.33 ± 1.53	39.33 ± 3.78	34.40 ± 3.97	36.20 ± 5.12	0.203
GGT, U/L	0.98 ± 0.27	1.11 ± 0.29	0.81 ± 0.20	0.86 ± 0.35	0.418
DBIL, umol/L	0.87 ± 0.10	0.96 ± 0.10	0.96 ± 0.09	0.98 ± 0.13	0.916
IBIL, umol/L	2.00 ± 0.43	2.29 ± 0.33	2.14 ± 0.40	1.96 ± 0.48	0.939
ALP, U/L	36.67 ± 8.38	27.75 ± 4.19	27.67 ± 2.88	24.50 ± 4.93	0.073
TBA, umol/L	1.42 ± 0.38	1.53 ± 0.50	1.66 ± 0.57	1.68 ± 1.19	0.995

A, dietary supplements 0% *D. officinale* in poodles (as control); B, dietary supplements 0.5% *D. officinale* in poodles; C, dietary supplements 1% *D. officinale* in poodles; D, dietary supplements 2% *D. officinale* in poodles; TC, total cholesterol; γ-IFN, interferon-γ; IgM, immunoglobulin M; GLU, glucose; TP, total protein; ALB, albumin; GLB, globulin; A/G, white globulin ratio; TBil, total bilirubin; ALT, alanine aminotransferase; AST, aspartate aminotransferase; GGT, glutamyl transferase; DBIL, direct bilirubin; IBIL, indirect bilirubin; ALP, alkaline phosphatase; TBA, total bile acids. Different letters (a, b, c) indicate statistical significance. Shoulder labels in peer data that did not contain the same letter indicated significant differences between these data *p* < 0.05, there was no significant difference between the same letters or no letters *p* > 0.05.

### 4.3 Serum antioxidant capacity

According to Table 4, the inclusion of *D. officinale* in the diet significantly influenced the activities of serum IgM, CAT, GSH-Px and T-AOC in poodles (*p* < 0.05). The highest IgM levels were recorded at 0.5 and 1% *D. officinale* supplementation, reaching 1.02 g/L and 1.04 g/L, respectively. The highest CAT levels were recorded at 0.5 and 1% supplementation, reaching 1.94 U/mL and 1.95 U/mL, respectively. However, a significant decreased in CAT content was observed with the addition of *D. officinale* at 2%. The peak GSH-Px activity was noted at 2% supplementation, achieving 1630.56 U/mL, while the highest T-AOC was found at 1% supplementation, reaching 7.12 U/mL. No significant effect on serum MDA content was detected (*p* > 0.05). These findings indicate that *D. officinale* enhances the immune system and antioxidant capacity in poodles.

**Table 4:** Effects of *D. officinale* on serum immunity and antioxidant status of poodles (*N* = 8).

Items	Groups				P value
	A	B	C	D	
γ-IFN, ng/L	8.83 ± 0.67	8.85 ± 2.26	8.96 ± 2.30	8.54 ± 2.43	0.997
Ig M, g/L	0.64 ± 0.10^b^	1.02 ± 0.10^a^	1.04 ± 0.09^a^	0.77 ± 0.10^ab^	0.012
CAT, U/mL	1.59 ± 0.20^b^	1.94 ± 0.16^a^	1.95 ± 0.29^a^	1.38 ± 0.09^b^	0.003
MDA, nmol/mL	7.30 ± 1.77	7.13 ± 1.81	6.46 ± 1.23	6.63 ± 1.68	0.826
GSH-Px, U/ml	1425.58 ± 53.58^b^	1536.21 ± 39.51^a^	1595.35 ± 21.48^a^	1630.56 ± 25.37^a^	0.004
T-AOC, U/mL	5.44 ± 0.32^b^	6.76 ± 0.23^a^	7.12 b ± 0.28^a^	6.45 b ± 0.30 ^a^	0.003

A, dietary supplements 0% *D. officinale* in poodles (as control); B, dietary supplements 0.5% *D. officinale* in poodles; C, dietary supplements 1% *D. officinale* in poodles; D, dietary supplements 2% *D. officinale* in poodles; CAT, catalase; MDA, malondialdehyde; GSH-Px, glutathione peroxidase; T-AOC, total antioxidant capacity. Different letters (a, b, c) indicate statistical significance. Shoulder labels in peer data that did not contain the same letter indicated significant differences between these data *p* < 0.05, there was no significant difference between the same letters or no letters *p* > 0.05.

### 4.4 Plasma metabolomics

#### 4.4.1 Plasma metabolite quantities and data quality assessment

**Figure 1:**
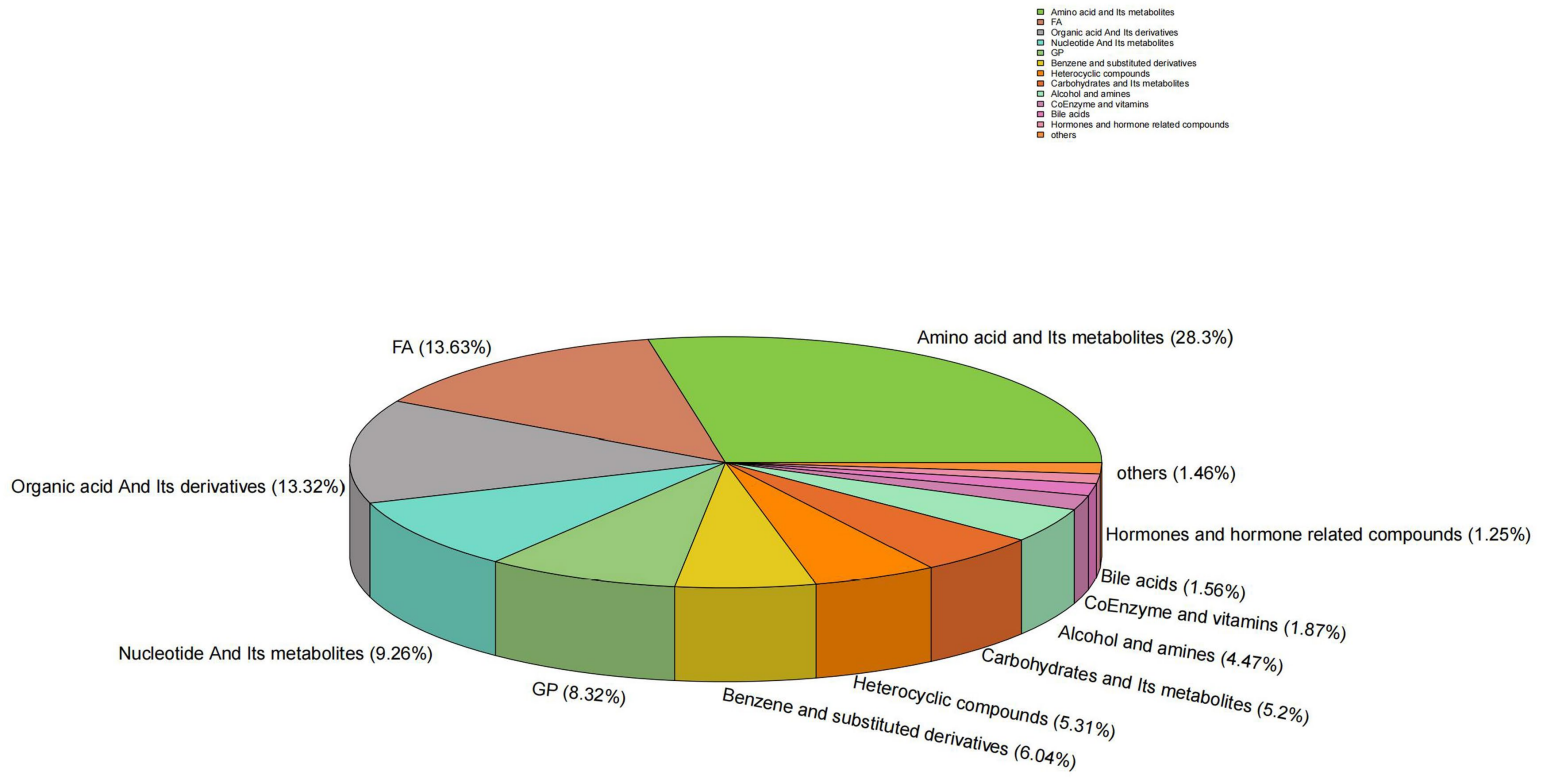
Circular diagram of metabolite class composition. Each color represents a category of metabolites, and the area of the color block indicates the proportion of that category.

**Figure 2:**
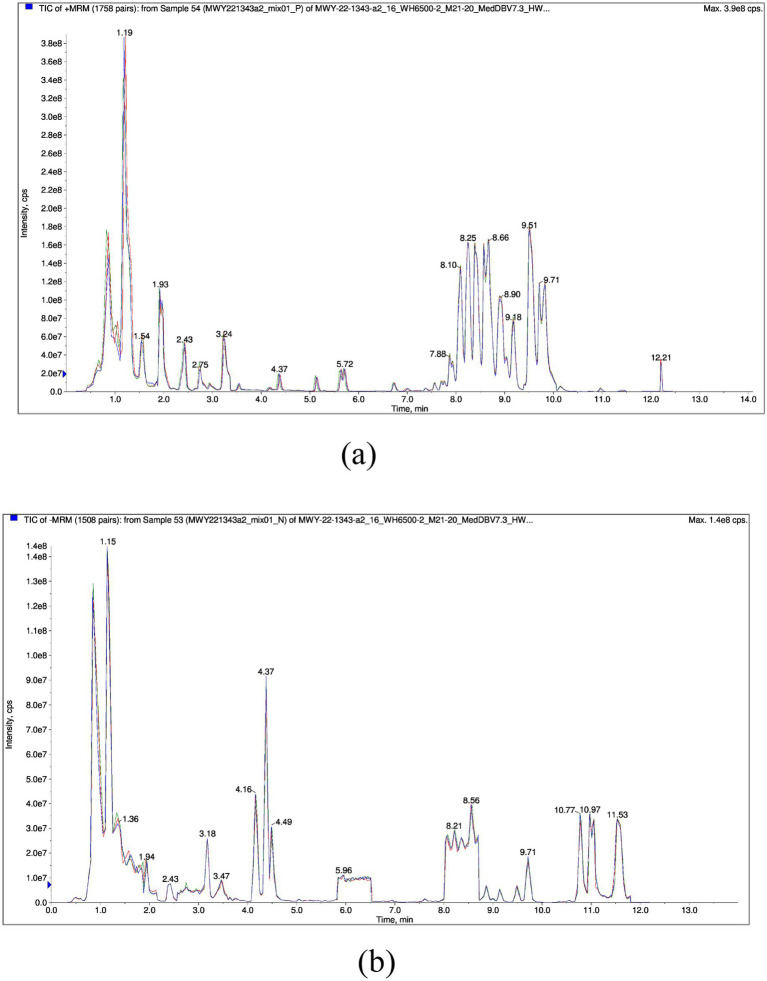
Overlapping total ion current (TIC) of QC sample mass spectrometry analysis. **(a)** Is Positive ion mode, **(b)** is Negative ion mode. The horizontal axis represents the retention time (Rt) of the metabolites, while the vertical axis represents the ion flow intensity of the ion detection (with intensity measured in cps, count per second).

A total of 961 metabolites were identified, with the classification results presented in Figure 1. Amino acids and their metabolites accounted for 28.30% of the total, while fatty acyls represented 13.63%. Organic acids and their derivatives, nucleotides and their metabolites, glycerophospholipids, and benzene and its derivatives comprised 13.32, 9.26, 8.32, and 6.0% of the total, respectively. The remaining 4.04% of the metabolites were identified as heterocyclic compounds, 5.31% as carbohydrates and their metabolic products, 4.47% as alcohols and amines, 1.87% as coenzymes and vitamins, 1.56% as bile acids, 1.25% as hormones and hormone-related compounds, 0.52% as tryptamines, cholines, and pigments, 0.42% as sphingolipids, 0.1% as aldehydes, ketones, and esters, 0.1% as glycerolipids, and 0.31% as other metabolites. To validate the precision of the analytical outcomes, the total ion current (TIC plots) of various quality control QC samples subjected to mass spectrometry detection were superimposed and presented. The results are illustrated in Figures 2a,b. The response intensities and retention times of the peaks exhibited significant overlap, demonstrating that the instrument exhibited high stability, reproducibility, and reliability.

#### 4.4.2 OPLS-DA analysis

**Figure 3:**
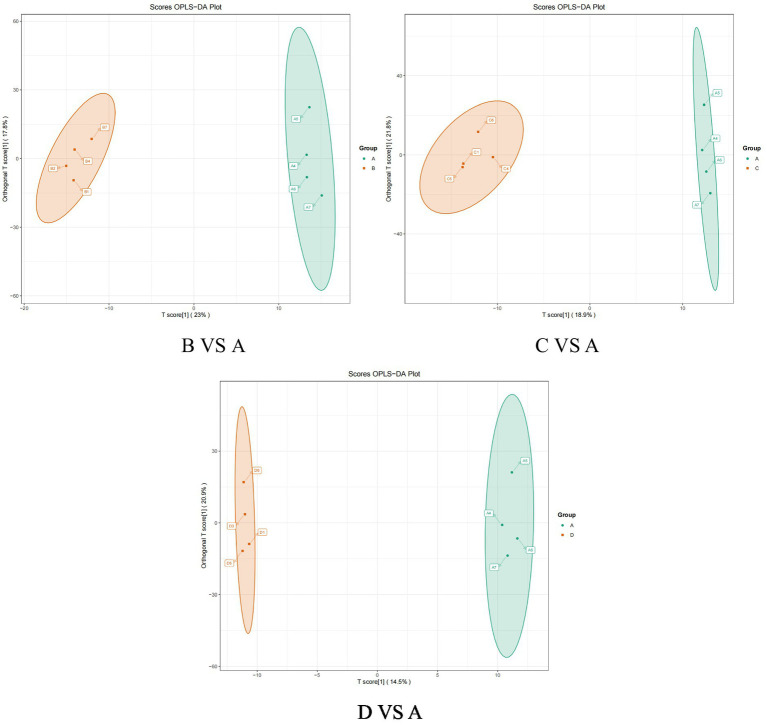
The orthogonal partial least squares-discriminant analysis (OPLS-DA) score plot between the control group and treatment group.

**Figure 4:**
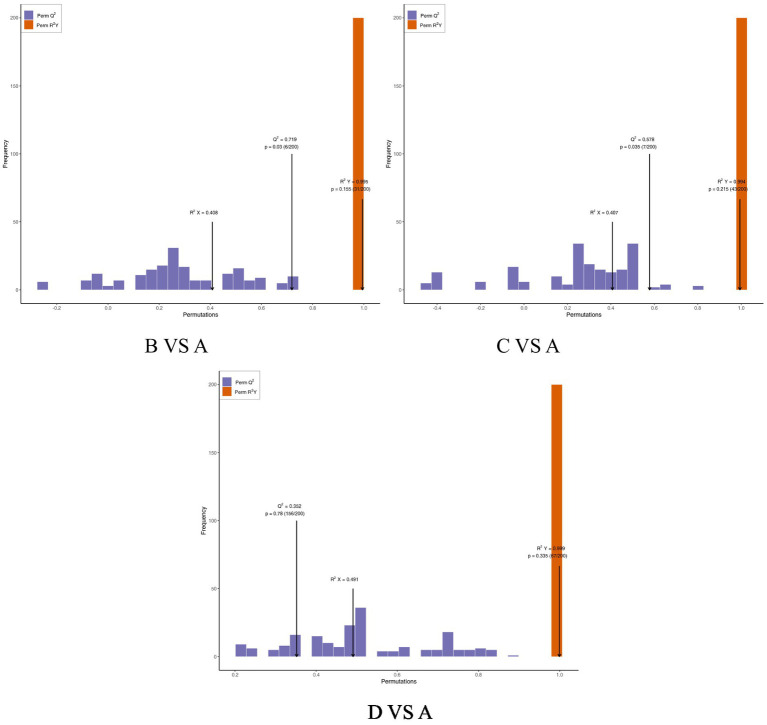
Validation of the OPLS-DA model between the control group and treatment group. *R*^2^X and *R*^2^Y represent the explanatory power of the constructed model for the X and Y matrices, respectively, while Q^2^ indicates the predictive ability of the model. The closer these three indicators are to 1, the more stable and reliable the model is. Q^2^ > 0.5 is considered to indicate an effective model.

OPLS-DA is an enhanced version of Partial Least Squares Discriminant Analysis (PLS-DA) that effectively filters out irrelevant information, thereby improving the model’s ability to differentiate between groups and enhancing its analytical power. As illustrated in the OPLS-DA score plot (Figure 3) and model validation plot (Figure 4), the model parameters R^2^Y > 0.9 and Q^2^ > 0.5 indicate that the OPLS-DA model is stable, reliable, and possesses strong predictive capability. Furthermore, a distinct separation is observed between the control group and each test group.

#### 4.4.3 Screening for differential metabolites

**Figure 5:**
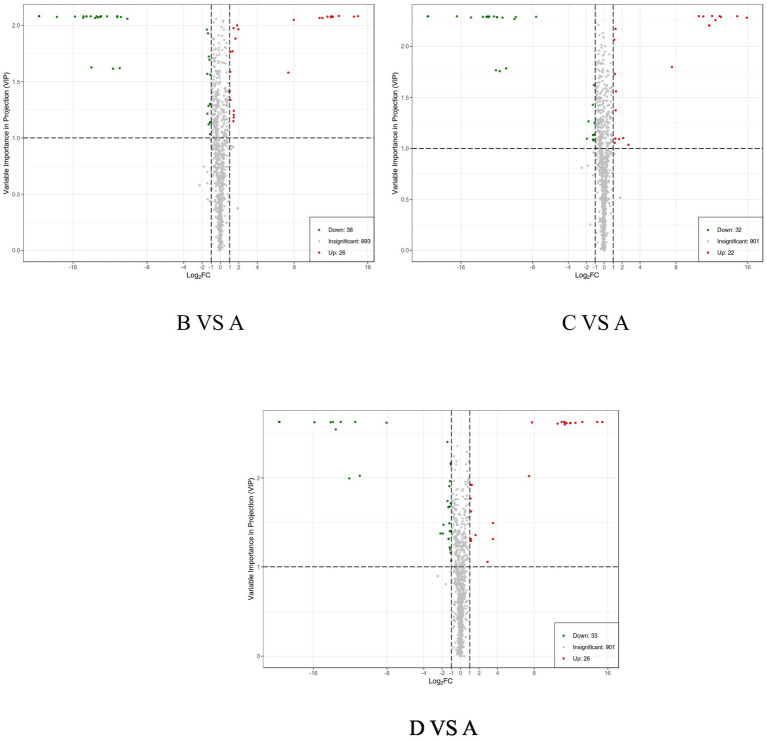
Volcano plot of differential metabolites. Each point represents a type of metabolite, with green points indicating down-regulated differential metabolites, red points indicating up-regulated differential metabolites, and gray points representing metabolites that were detected but not significantly different.

Differentially expressed metabolites can be identified using the OPLS-DA model, focusing on VI*p* values (VIP ≥ 1) in conjunction with differential fold change values (Fold Change FC ≥ 2 or FC ≤ 0.5). As detailed in the statistical table of differential metabolites (Table 5) and illustrated in the volcano plot (Figure 5), a total of 64 metabolites with significant differences were identified in group B compared to groupA, comprising 26 upregulated and 38 downregulated metabolites. Similarly, 54 metabolites with significant differences were identified in group C compared to groupA, including 22 upregulated and 32 downregulated metabolites. In groupD compared to group A, a total of 59 metabolites with significant differences were identified, with 26 upregulated and 33 downregulated metabolites.

**Table 5:** Statistical analysis of the number of differential metabolites between the control group and each experimental group (*N* = 4).

Treatments	Total sig metabolites	Down regulated	Up regulated
B VS A	64	38	26
C VS A	54	32	22
D VS A	59	33	26

A, dietary supplements 0% *D. officinale* in poodles (as control); B, dietary supplements 0.5% *D. officinale* in poodles; C, dietary supplements 1% *D. officinale* in poodles; D, dietary supplements 2% *D. officinale* in poodles.

#### 4.4.4 Functional annotation and enrichment analysis of differential metabolites in KEGG

The KEGG database offers an integrated network for the study of genes, expression data, and metabolite concentrations. Conducting pathway enrichment analysis on differential metabolites facilitates the understanding of the mechanisms that drive their alterations within specific pathways. The identified differential metabolites can be annotated and analyzed for pathway enrichment utilizing the KEGG database.

**Figure 6:**
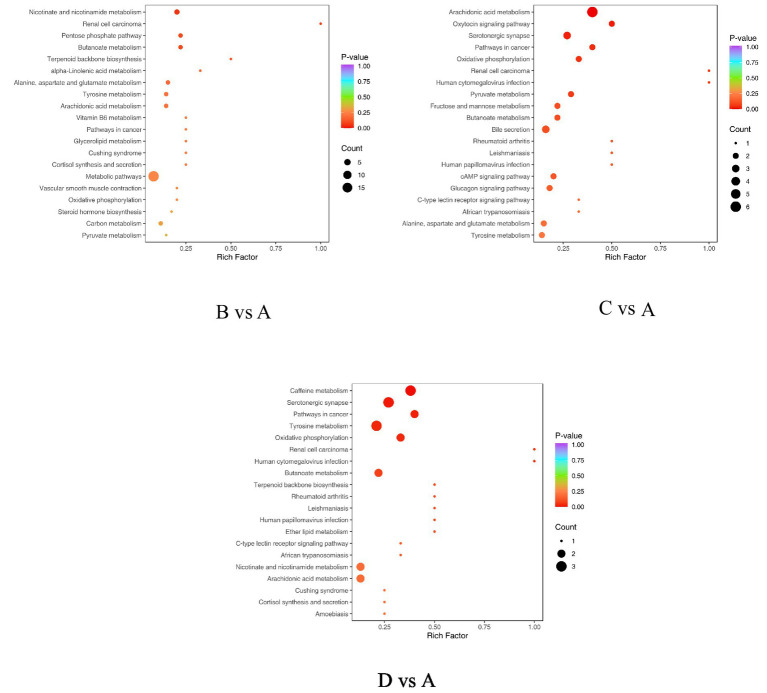
Enrichment plot of differential metabolites in KEGG. The horizontal axis represents the Rich Factor corresponding to each pathway, while the vertical axis shows the pathway names (sorted by *p*-value). The color of the points reflects the size of the p-value, with red indicating more significant enrichment. The size of the points represents the number of differential metabolites enriched.

As illustrated in Table 6 and Figure 6, the differential metabolites between Group B and Group A are primarily annotated and enriched in the following pathways: arachidonic acid metabolism (ko00590), nicotinate and nicotinamide metabolism (ko00760), and butanoate metabolism (ko00650). The involved key metabolites include fumaric acid and thromboxane B2. In the comparison between Group C and Group A, the differential metabolites are mainly annotated and enriched in arachidonic acid metabolism (ko00590), pyruvate metabolism (ko00620), butanoate metabolism (ko00650), alanine, aspartate, and glutamate metabolism (ko00250), and tyrosine metabolism (ko00350). The major metabolites identified are 8-iso-prostaglandin F2α, fumaric acid, L-lactic acid, prostaglandin E2, and thromboxane B2. For the comparison between GroupD and Group A, the differential metabolites are primarily annotated and enriched in arachidonic acid metabolism (ko00590), nicotinate and nicotinamide metabolism (ko00760), butanoate metabolism (ko00650), tyrosine metabolism (ko00350), and caffeine metabolism (ko00232). The involved key metabolites in this comparison include 1,7-dimethylxanthine, 5-acetylamino-6-amino-3-methyluracil, 5-hydroxyindole-3-acetic acid, fumaric acid, prostaglandin E2, p-hydroxyphenylacetic acid, thromboxane B2, and theophylline.

**Table 6:** Enrichment analysis of KEGG metabolic pathways of differential metabolites (*N* = 4).

Treatments	Metabolites	VIP	FC	Type	Pathway ID
B vs. A	Fumaric Acid	2.08	1.18E-06	down	ko00650,ko00760,ko01100
	Thromboxane B2	1.34	2.09	up	ko00590,ko01100
C vs. A	8-iso Prostaglandin F2α	1.08	0.43	down	ko00590
	Fumaric Acid	2.29	1.18E-06	down	ko00250,ko00350,ko00650
	L-Lactic Acid	1.09	3.12	up	ko00620,ko01100
	Prostaglandin E2	2.20	3351.30	up	ko00590,ko01100
	Thromboxane B2	1.04	6.55	up	ko00590,ko01100,ko04726
D vs. A	1,7-Dimethylxanthine	1.32	0.41	down	ko00232,ko01100
	5-Acetylamino-6-amino-3-methyluracil	2.17	0.47	down	ko00232
	5-Hydroxyindole-3-Acetic Acid	2.62	5.61E-05	down	ko00380,ko01100,ko04726
	Fumaric Acid	2.63	1.18	down	ko00350,ko00650,ko00760,ko01100
	Prostaglandin E2	2.63	2488.38	up	ko00590,ko01100
	P–Hydroxyphenyl Acetic Acid	1.68	0.43	down	ko00350,ko01100
	Thromboxane B2	1.36	3.10	up	ko00590,ko01100
	Theophylline	1.32	0.41	down	ko00232,ko01100

A, dietary supplements 0% *D. officinale* in poodles (as control); B, dietary supplements 0.5% *D. officinale* in poodles; C, dietary supplements 1% *D. officinale* in poodles; D, dietary supplements 2% *D. officinale* in poodles; VIP, variable importance in projection; FC, fold change.

#### 4.4.5 Correlation analysis between plasma metabolites and blood biochemical indicators and antioxidant indicators

**Figure 7:**
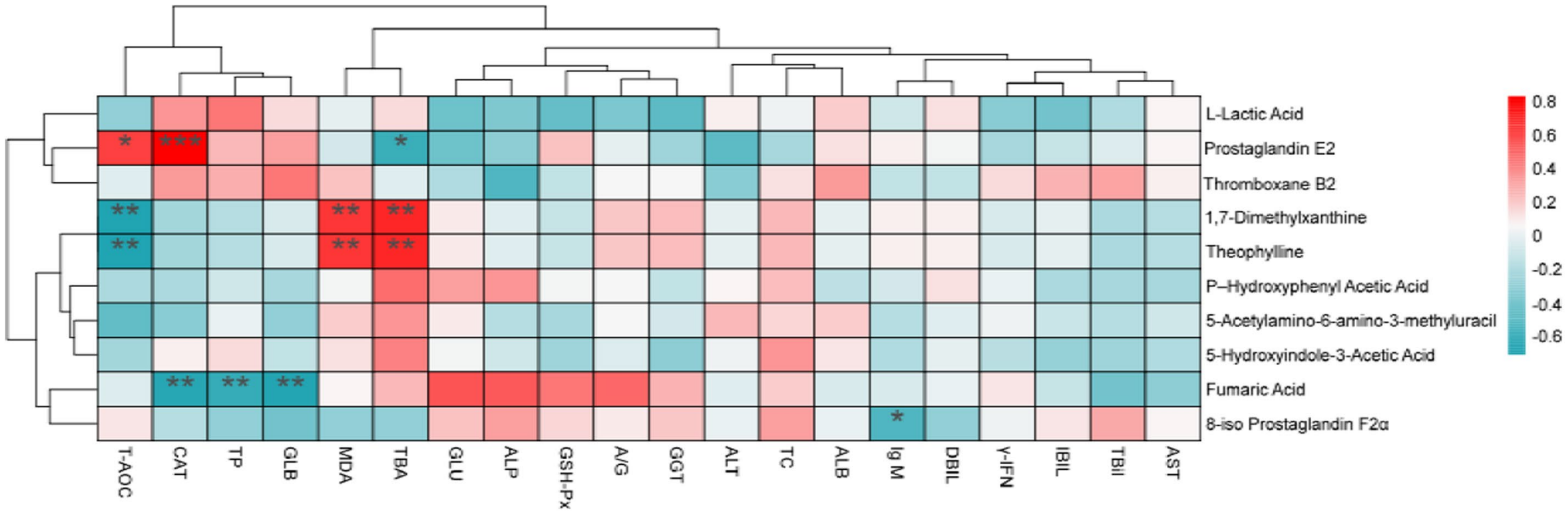
Statistical Spearman’s correlations between the blood metabolites and blood biochemical and index antioxidant index. TC, Total cholesterol; *γ*-IFN, Interferon-γ; IgM, immunoglobulin M; GLU, glucose; TP, total protein; ALB, albumin; GLB, globulin; A/G, white globulin ratio; TBil, total bilirubin; ALT, alanine aminotransferase; AST, aspartate aminotransferase; GGT, glutamyl transferase; DBIL, direct bilirubin; IBIL, indirect bilirubin; ALP, alkaline phosphatase; TBA, total bile acids; CAT, catalase; MDA, malondialdehyde; GSH-Px, glutathione peroxidase; T-AOC, total antioxidant capacity. *p < 0.05, **p < 0.01.

To investigate the relationship between changes in blood biochemical indicators and antioxidants with alterations in blood metabolites, we conducted a Spearman correlation analysis (Figure 7). Prostaglandin E2 exhibited a positive correlation with CAT and T-AOC, while demonstrating a negative correlation with TBA. Furthermore, 1,7-Dimethylxanthine displayed a negative correlation with T-AOC and a positive correlation with MDA and TBA. Fumaric acid found to be negatively correlated with CAT, TP, and GLB, whereas 8-iso-prostaglandin F2α showed a negative correlation with IgM.

## 5 Discussion

### 5.1 The effect of *Dendrobium officinale* on nutrients digestibility in poodles

Apparent nutrient digestibility directly reflects dietary utilization efficiency in canines. Contemporary pharmacological studies have revealed that *Dendrobium* has significant gastrointestinal regulatory functions, which can enhance gastric emptying capacity, improved intestinal peristalsis, and promoted digestive-excretory processes (21). Investigations using murine models further demonstrate that *Dendrobium* polysaccharides induce notable intestinal modifications: they stimulate the production of secretory immunoglobulin A (sIgA), increase the villus height/crypt depth ratios in the small intestine mucosa, and expand the surfaces available for nutrient absorption (22). However, this study did not find any significant affecte of *D. officinale* on the digestibility of nutrients in poodles, which may be attributed to species differences.

### 5.2 The effect of *Dendrobium officinale* on blood biochemical parameters in poodles

Serum biochemical indices in animal organisms serve as crucial biomarkers that reflect both physiological and metabolic status, while also providing insights into immune competence (23). Immunoglobulins, produced by plasma cells differentiated from B lymphocytes following antigen stimulation, generating as specific antibodies capable of targeted antigen binding. Notably, IgM the largest molecular weight antibody class, is predominantly synthesized and secreted by splenic and lymph node plasma cells (24). Structurally, total serum proteins include two principal fractions: albumin and globulin (25). Serum albumin plays a crucial role in maintaining osmotic pressure, ensuring the proper distribution of nutrients, and both organic and inorganic ions, while facilitating detoxification processes (26). Serum globulins are primarily composed of complement proteins and immunoglobulins, which facilitate complement activation, antigen binding, and immune regulation (27). Emerging pharmacological evidence demonstrates the immunomodulatory potential of Dendrobium phytoconstituents. Chen et al. (9) and Fan et al. (11) systematically validated that bioactive compounds derived from Dendrobium species significantly enhance immune responses through multiple pathways. Murine experimental models have revealed three distinct immunostimulatory mechanisms of Dendrobium: (1) activation of splenic lymphocyte differentiation/proliferation, (2) augmentation of peritoneal macrophage phagocytic activity, and (3) elevation of serum immunoglobulin levels to strengthen humoral immunity (16, 17). This immunoenhancement profile has been cross-species validated in avian models, with *D. officinale* extracts demonstrating comparable immune-boosting efficacy in poultry through analogous mechanisms (28).

The findings of this study revealed that dietary supplementation with *D. officinale* significantly elevated serum levels of IgM, total protein, and globulin in canines. These results further demonstrate that *D. officinale* enhances the host’s capacity to resist exogenous stimuli and pathogens, thereby improving immune function. This immunomodulatory effect may be attributed to the polysaccharides in *D. officinale*, which regulate immune responses through multi-target mechanisms, including promoting immune cell proliferation and differentiation, activating the complement system, and modulating cytokine release (15). Notably, high-dose Dendrobium supplementation (2%) exhibited immunosuppressive effects, indicating a non-linear positive correlation between *Dendrobium* concentration and immune competence, characterized by a distinct threshold characteristic. This phenomenon may be attributed to a dose-dependent biphasic regulatory mechanism. Previous studies have reported that certain phytochemical constituents (flavonoids and alkaloids) at elevated concentrations may induce oxidative stress or suppress immunocyte proliferation, consequently compromising immunoenhancing efficacy (29). In the present study, optimal immunomodulatory efficacy was observed at *D. officinale* supplementation levels of 0.5 to 1%.

### 5.3 The effect of *Dendrobium officinale* on antioxidant capacity in poodles

The antioxidant system serves as a critical defense mechanism for organisms, enabling the scavenging of excess reactive oxygen species (ROS) and mitigating oxidative damage (30). CAT, a terminal oxidase, specifically catalyzes the decomposition of hydrogen peroxide (H_2_O_2_) into water and molecular oxygen, thereby reducing the cytotoxicity associated with H_2_O_2_ accumulation (31). As a pivotal antioxidant enzyme, GSH-Px primarily neutralizes hydrogen peroxide and lipid hydroperoxides through redox reactions (32). T-AOC quantitatively reflects the systemic balance between oxidative stress and antioxidant defenses within biological systems (33). MDA is a key byproduct of membrane lipid peroxidation, serving as a biomarker for evaluating free radical-mediated cellular injury, with its concentration positively correlating with the severity of oxidative damage (34). Research has demonstrated that polysaccharides derived from *D. officinale* exhibit antioxidant properties through the modulation of the antioxidant enzyme system (35). Qi et al. (36) revealed that *Dendrobium* candidum polysaccharides demonstrate protective effects against aortic oxidative stress in atherosclerotic mice by enhancing serum SOD and GSH-P_X,_ while concurrently reducing MDA concentrations. Liang et al. (13) found that both the juice of *D. officinale* and its polysaccharide components effectively elevated serum glutathione peroxidase activity and total antioxidant capacity in murine models, while also exhibiting anti-aging properties. This study demonstrated that incorporating *D. officinale* into dog food significantly enhances serum CAT and GSH-Px levels, as well as total antioxidant capacity, consistent with previous research findings. These observed effects are likely attributable to the bioactive constituents of *D. officinale*, particularly its rich profile of natural antioxidants including flavonoids and phenolic compounds (37). These phytochemicals have been extensively documented as potent antioxidants, effective free radical scavengers, and anti-aging agents through multiple mechanistic pathways (37, 38). Furthermore, accumulating evidence indicated that *D. officinale* polysaccharides upregulate the expression of the nuclear factor erythroid 2-related factor 2 (Nrf2) antioxidant gene (10, 12). This transcriptional activation subsequently enhances the expression of Glutamate-cysteine ligase catalytic subunit (GCLC) and Glutamate-cysteine ligase modifier subunit (GCLM), which are key enzymes in glutathione biosynthesis. The resultant elevation in antioxidant enzyme levels ultimately enhances systemic antioxidant capacity in biological systems (10, 12).

### 5.4 The impact of *Dendrobium officinale* in poodles blood metabolomics

Metabolomics data are characterized by their high-dimensional and large-scale nature. By integrating univariate and multivariate statistical analyses and examining data from multiple perspectives based on its characteristics, differential metabolites can be accurately identified (39). This approach holds unique advantages and plays a crucial role in animal nutrition research (40). Wang (18). found that the polysaccharides in *D. officinale* can alleviate subacute alcoholic liver injury in mice by modulating glucose metabolism, amino acid metabolism, and lipid metabolism pathways. The present study revealed that *D. officinale* significantly affects the serum concentrations of ten metabolites in poodles, including Fumaric Acid, 8-isoprostane F2a, L-lactic acid, Prostaglandin E2, Thromboxane B2, 1,7-Dimethylxanthine, 5-Acetylamino-6-amino-3-methyluracil, 5-Hydroxyindole-3-Acetic Acid, Hydroxyphenylacetic acid, and Theophylline in plasma (*p* < 0.05). As intermediate metabolites or substrates, these compounds participate in various metabolic pathways, primarily enriched in arachidonic acid metabolism, caffeine metabolism, pyruvate metabolism, and amino acid metabolism.

The arachidonic acid metabolic pathway produces various eicosanoids that positively influence the body’s antioxidant capacity and immune response (41, 42). Prostaglandin E2, a member of the eicosanoid family, binds to the Prostaglandin E Receptor 2, thereby regulateing the antioxidant signaling pathway in macrophages. This interaction reduces free radical production and enhances the antioxidant capacity of macrophages, leading to anti-inflammatory effects (41, 42). Additionally, 8-iso Prostaglandin F2α, a prostaglandin derivative formed through free radical-catalyzed non-enzymatic peroxidation of arachidonic acid, exhibits a positive correlation with inflammatory factors levels in the body, serving as a marker for oxidative stress. Elevated levels of 8-iso Prostaglandin F2α indicate increased oxidative stress and a corresponding decline in immune function (43). In this study, the incorporation of *D. officinale* into poodle food led to a significant increase in plasma levels of Prostaglandin E2 and a notable decrease in 8-iso Prostaglandin F2α. Spearman correlation analysis further indicated that Prostaglandin E2 was positively correlated with antioxidant capacity, whereas 8-iso Prostaglandin F2α exhibited a negative correlation with the immune marker IgM. These findings suggest that *D. officinale* modulates arachidonic acid metabolism, reduces oxidative stress, and consequently enhances both antioxidant capacity and immune function. Liu et al. (44) employed metabolomics techniques to investigate the effects of *D. officinale*, demonstrating its capability to regulate arachidonic acid metabolism and restore lipid metabolic balance within the body.

The biotransformation of caffeine involves complex hepatic metabolic processes mediated by multiple enzyme systems, predominantly yielding various purine derivatives and uric acid metabolites (45). Specifically, 1,7-dimethylxanthine and theophylline as xanthine derivatives and end-products of purine metabolism, are ubiquitously distributed in human biological fluids (45). As the primary degradation product of caffeine through N-demethylation, theophylline undergoes further catabolism to 3-methylxanthine or 1-methylxanthine. This methylxanthine exhibits pharmacological activities including smooth muscle relaxation and bronchodilation, with emerging evidence supporting its anti-inflammatory and immunomodulatory properties (46). In the present study, dietary supplementation with *D. officinale* at concentrations of 0.5 to1% demonstrated no significant alteration in systemic theophylline levels. However, a 2% supplementation dose resulted in a marked reduction of serum theophylline concentrations in poodles (*p* < 0.05), suggesting potential adverse effects on methylxanthine homeostasis at higher dosage thresholds.

L-lactate, the principal metabolic product of the pyruvate pathway, serves as a critical energy shuttle in mammalian systems. In addition to its biosynthetic roles, it functions as a signaling molecule that regulates hormone release and the activity of various enzymes in the body (47). Studies have indicated that a moderate increase in circulating L-lactic acid levels can improve metabolic health (48). Tauffenberger et al. (49) demonstrated that L-lactate induces controlled generation of reactive oxygen species (ROS), which activates cellular defense systems, thereby providing protection against oxidative damage. The present study found that supplementation with *D. officinale* at a concentration of 1% significantly increased serum L-lactate levels in poodles, contributing to the enhancement of the body’s antioxidant capacity.

P-hydroxyphenylacetic acid, a principal metabolite in tyrosine catabolism, serves as the biosynthetic precursor for p-hydroxyphenylpyruvate. This intermediate undergoes enzymatic conversion via homogentisate to ultimately yield fumarate in the tricarboxylic acid (TCA) cycle (50). Evidence indicates that endogenous fumaric acid reduce the oxidative capacity of the body by binding to glutathione (51). Cheng et al. (52) elucidated that fumarate exerts immunomodulatory effects by directly inactivating the tyrosine-protein kinase LYN, subsequently suppressing B-cell activation and functional responses. In this study, *D. officinale* supplementation significantly reduced serum concentrations of both Phydroxyphenyl Acetic Acid and fumaric acid in poodles. Correlation analysis further revealed a negative correlation between fumaric acid and CAT levels in the body. This suggests that the observed enhancement in antioxidant capacity may be mechanistically linked to fumarate homeostasis modulation through Dendrobium intervention.

## 6 Conclusion

The inclusion of *D. officinale* in the diet did not influence the nutritional digestion and metabolism of dogs. However, it was observed that it can significantly enhance the immunity and antioxidant capacity of poodles, with the most pronounced effects occurring at an addition level of 0.5 to 1%. Furthermore, blood metabolomic analysis indicated that *D. officinale* may modulate the antioxidant capacity and immunity of poodles through the metabolism of biotaenoic acid, caffeine, pyruvate and amino acids.

## Data Availability

The data presented in the study are deposited in the NGDC (National Genmics Data Center) repository, accession number “OMIX010434”.
